# 非小细胞肺癌患者*hOGG1*基因启动子区域突变的研究

**DOI:** 10.3779/j.issn.1009-3419.2011.03.04

**Published:** 2011-03-20

**Authors:** 霞 刘, 军 赵, 仍允 刘, 哲 雷, 嘉琮 尤, 清华 周, 洪涛 张

**Affiliations:** 1 215123 苏州，苏州大学癌症分子遗传学实验室 Soochow University Laboratory of Cancer Molecular Genetics, Medical College of Soochow University, Suzhou 215123, China; 2 300052 天津，天津医科大学总医院，天津市肺癌研究所，天津市肺癌转移与肿瘤微环境重点实验室 Tianjin Key Laboratory of Lung Cancer Metastasis and Tumor Microenvironment, Tianjin Lung Cancer Institute, Tianjin Medical University General Hospital, Tianjin 300052, China

**Keywords:** 肺肿瘤, PCR-SSCP, *hOGG1*基因, 突变, Lung neoplasms, PCR-SSCP, *hOGG1* gene, Mutation

## Abstract

**背景与目的:**

8-羟基鸟嘌呤DNA糖苷酶（8-hydroxygumine DNA glycosylase 1, OGG1）是一种DNA修复酶，可以从DNA切除修复8-羟基鸟嘌呤（8-dihydro-8-oxoguanine, 8-OH-G）。人类*OGG1*基因（*hOGG1*）的多态性可能会改变酶的活性而影响个体修复损伤DNA的能力，促进癌变。然而，*hOGG1*基因启动子区域的突变与非小细胞肺癌（non-small cell lung cancer, NSCLC）的关系尚不明晰。我们拟探讨*hOGG1*基因启动子区域的突变与NSCLC发生发展的潜在关系。

**方法:**

选取苏州大学附属第一医院2003年1月-2005年12月新鲜手术切除的40例NSCLC组织标本，采用PCR-SSCP和直接测序的方法检测NSCLC及其对应的癌旁组织中*hOGG1*基因启动子区域的突变。

**结果:**

在40例NSCLC患者中未发现*hOGG1*基因启动子区域的异常突变，但发现单核苷酸多态位点rs159153与TNM分期明显相关（*P*=0.008）；同时发现吸烟者中淋巴结转移率明显较低（*P*=0.034）。

**结论:**

单核苷酸多态位点rs159153和吸烟史可能对NSCLC的侵袭和转移潜在性提供预测。

## Abstract (en)

**Background and objective:**

8-hydroxygumine DNA glycosylase 1 (OGG1) is a DNA repair enzyme, which can repair damaged DNA by excising 8-dihydro-8-oxoguanine (8-OH-G). Polymorphisms in human *OGG1* gene (*hOGG1*) may alter glycosylase activity, thereby affects its repair to the damaged DNA, resulting in contribution to carcinogenesis. However, an association of genetic variants of *hOGG1* promoter with non-small cell lung cancer (NSCLC) remains unclear. The present study aims to explore whether there are mutations in the promoter region of *hOGG1* and the association of the potential genetic variants with NSCLC.

**Methods:**

Forty lung cancer patients were enrolled from January, 2003 to December, 2005 in the first affiliated hospital of Soochow University. PCR-SSCP followed by direct sequencing were performed to detect mutations within the promoter region of the *hOGG1* gene in NSCLC and corresponding paracancerous lung tissues.

**Results:**

No abnormal mutation was found in the promoter region of the *hOGG1* gene in 40 patients with NSCLC. However, a SNP rs159153 in *hOGG1* was significantly associated with higher TNM stage (*P*=0.008). Moreover, lower frequency of lymph node metastasis was observed in smoker patients with NSCLC (*P*=0.034).

**Conclusion:**

The SNP rs159153 in the promoter region of the *hOGG1* gene and smoking history may effectively forebode the aggressiveness and metastatic potential of NSCLC.

正常的氧化代谢和环境压力都可以对持续暴露其中的细胞DNA造成损伤，从而影响基因组的稳定性。生物演化出不同的DNA修复途径对不同的DNA损伤进行修复，其中最主要的途径就是通过特定的DNA糖基化酶对错配碱基进行识别和切除修复。8-羟基鸟嘌呤（8-dihydro-8-oxoguanine, 8-OH-G）是一种主要的DNA氧化损伤，此种异常碱基不能阻止DNA链延伸，在复制时由于空间构象改变使该碱基优先与腺嘌呤配对，可导致G:C→T:A颠换突变^[1]^，若不及时修复可导致DNA突变的积累。因此，该突变与肿瘤的发生发展、机体细胞的老化及某些退行性疾病均有密切关系^[2-4]^。体内特异识别8-OH-G并将其切除修复的酶被称为8-羟基鸟嘌呤DNA糖苷酶（8-hydroxygumine DNA glycosylase, OGG1），通常称人类OGG1为hOGG1。该基因位于人染色体3p26.2，起始密码子ATG位于第1外显子中。根据对第7和第8外显子的不同选择，*hOGG1*基因可以选择性拼接产生两大类hOGG1蛋白，α型和β型^[2]^。α-hOGG1蛋白含345个氨基酸，分子量为39 kDa，有核定位信号；β-hOGG1蛋白含424个氨基酸，分子量为47 kDa，定位于线粒体。

由于许多内源性和外源性致癌物可产生8-OH-G，所以修复8-OH-G的能力与个体对致癌物作用的敏感性密切相关。无论*hOGG1*基因突变或缺失，都可能使细胞修复8-OH-G的能力降低或丧失，从而使此种个体罹患肿瘤的风险增高。关于*hOGG1*基因的突变和多态性与DNA氧化损伤修复能力及肿瘤易感性的关系已经引起一些研究者的关注^[5-7]^。本研究采用单链构象多态性（single-strand conformation polymorphism, SSCP）分析检测部分非小细胞肺癌（non-small cell lung cancer, NSCLC）患者癌组织和癌旁正常组织中*hOGG1*基因的启动子区域的突变，研究其与NSCLC发生、发展、分化及转移的关系。

## 1 材料与方法

### 1.1 临床样本

原发性NSCLC 40例，为苏州大学附属第一医院2003年1月-2005年12月新鲜手术切除标本，男28例，女12例，年龄37岁-79岁，中位年龄60岁。患者术前均未接受放、化疗。肿瘤组织均取自肿瘤中心非坏死部位，癌旁组织取自肿瘤旁约5 cm正常肺组织，组织标本切除后30 min内入液氮冻存。所有病例均有病理学诊断，其中腺癌16例，鳞癌15例，腺鳞癌1例，大细胞癌6例，不典型类癌2例；有淋巴结转移19例，无淋巴结转移21例。Ⅰ期14例，Ⅱ期18例，Ⅲ期7例，Ⅳ期1例。

### 1.2 DNA的抽提

酚-氯仿抽提法抽提DNA。

### 1.3 PCR扩增

由Primer Premier 5.0软件设计引物。引物均由上海生工生物工程技术服务有限公司合成，引物序列见表 1。反应体系25 μL，94 ℃预变性5 min，94 ℃变性45 s，退火温度30 s，72 ℃延伸30 s，35个循环，72 ℃后延伸10 min。PCR产物2%琼脂糖凝胶电泳，EB染色紫外凝胶成像分析系统拍照鉴定。

**1:** hOGG1启动子区PCR引物及SSCP条件 PCR primers used for the promoter of hOGG1 and SSCP conditions

No.		Primer sequence (5’→ 3’)	Product length	Incubate temp (T)	SSCP conditions	
					Concentration of PAGE gel	Electrophoresis time
1	FP	TCCCTTCTCTTTCCCATAGATTCC	209 bp	56.0 ℃	8%	4.5 h
	RP	CATGCACAGCAGTCAGCAACC				
2	FP	AGTGGTTGCTGACTGCTGTGC	257 bp	57.0 ℃	8%	5 h
	RP	CAGACCTGGGTTCCAATTCTAGC				
3	FP	AGAGCTAGAATTGGAACCCAGG	223 bp	54.5 ℃	8%	5 h
	RP	GGTGAGCAAAGAGCAACCG				
4	FP	ACAGAGCTGGGATGGAGACG	253 bp	58.0 ℃	8%	5 h
	RP	AGAGACCTAGCGGAGTGTACCG				
5	FP	CTAGGTCTCTGGCTGGGACACAAGG	273 bp	61.5 ℃	8%	5 h
	RP	CGGGTTCAAGCGATTCTCCTGC				
6	FP	AGGAGAATCGCTTGAACCCG	265 bp	58.6 ℃	8%	5 h
	RP	TCTAGTCGCCTGGAGTAGGAGG				
7	FP	ATCCATCCTCCTACTCCAGGCG	151 bp	58.0 ℃	8%	4 h
	RP	CATACTCCATCTGCTCATGCTTTCC				
8	FP	CCGAGGAAAGCATGAGCAGATGG	247 bp	59.1 ℃	6%	5 h
	RP	AACCCTGTCTTCACCAAGAACCGG				
9	FP	TGGTGAAGACAGGGTTCGTGGG	305 bp	60.6 ℃	8%	5.5 h
	RP	TAGCCAATCCGAGGAGGAGGTAGG				
10	FP	GGCTACCTCTAGGTGAAATGAGCGG	272 bp	61.0 ℃	8%	5.5 h
	RP	CCCACACGGTGCTGTTTAACAACC				
11	FP	ACAGGGAAGGTTGTTAAACAGCACC	266 bp	59.0 ℃	8%	5 h
	RP	CCGGAAAGATTGTCCAGAAGGC				
SSCP: single-strand conformation polymorphism; FP: forward primer; RP: reverse primer.						

### 1.4 SSCP分析

SSCP条件见表 1，非变性聚丙烯酰胺凝胶浓度和电泳时间根据产物长度及电泳条带结果进行调节。取10 μL PCR产物，加入10 μL变性剂（95%甲酰胺，0.05%溴酚蓝，0.05%二甲苯青，1×TBE），煮沸5 min，取出立刻放入冰浴中2 min以上，全部上样，8 ℃，20 W恒功率电泳5 h左右，取下凝胶，将其浸在含0.5 μg/mL溴化锭的1×TBE缓冲液中染色10 min-15 min，在紫外凝胶成像分析系统中拍照分析。

### 1.5 统计学分析

采用SPSS软件对数据进行小样本*Fisher*确切概率分析，*P* < 0.05为有统计学差异。

## 2 结果

### 2.1 *hOGG1*启动子区域的SSCP和DNA测序分析

40例NSCLC患者样本中只有第2号引物和第11号引物出现癌和癌旁对应一致的异常条带。2号引物有9例样本出现3带型（图 1），经测序表明是单核苷酸多态（single nucleotide polymorphism, SNP）-1096T/C（rs159153）；11号引物产物出现1例4带型和2例3带型两种电泳条带（图 2），测序结果表明为位于第一外显子的5’UTR区的SNP位点-23A/G（rs1801129）和-18G/T（rs1801126）。

**1:**
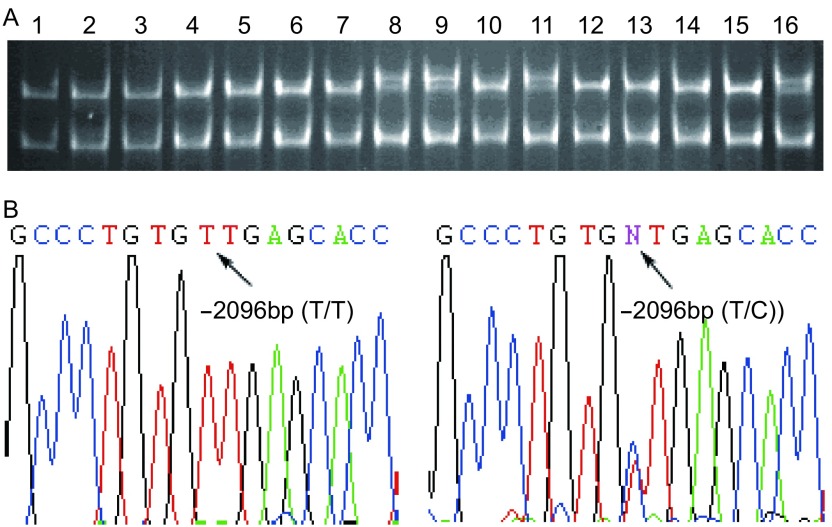
*hOGG1*第2对引物PCR扩增产物的SSCP和DNA测序。A：SSCP分析显示：8、9、11、16号样本呈三条带，其余为两条带；B：两条带PCR产物测序结果显示在-2096 bp处的基因型为T/T，三条带为T/C SSCP analysis and DNA sequencing for PCR product amplified by No.2 pair of primers. A: SSCP analysis shows an altered pattern with three bands in samples 8, 9, 11 and 16, and a normal pattern with two bands in other samples; B: DNA sequencing reveals that samples with three bands have T/C allele and those with two bands have T/T allele at the site -2096 bp

**2:**
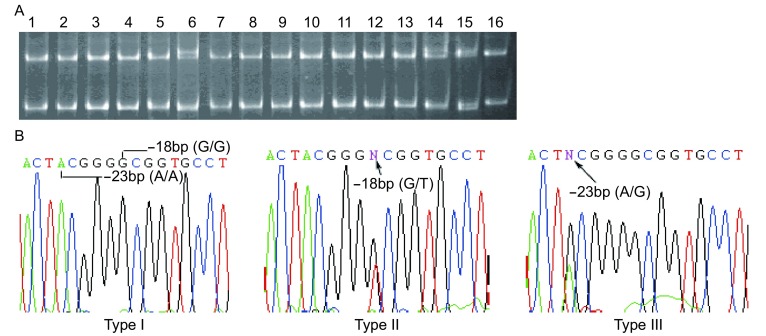
*hOGG1*第11对引物PCR扩增产物的SSCP和DNA测序。A：SSCP分析显示：6、14号样本呈三条带，15号四条带，其余为两条带；B：两条带PCR产物测序结果显示在-18 bp处的基因型为G/G，三条带为G/T；两条带PCR产物测序结果显示在-23 bp处的基因型为A/A，四条带为A/G SSCP analysis and DNA sequencing for PCR product amplified by No.11 pair of primers. A: SSCP analysis shows altered patterns with three bands in samples 6, 14 and four bands in sample 15, and a normal pattern with two bands in other samples; B: DNA sequencing reveals that samples with three bands have G/T allele and those with two bands have G/G allele at the site -18 bp, and the sample with four bands have A/G allele and those with two bands have A/A allele at the site -23 bp

### 2.2 NSCLC患者中rs159153多态基因型与临床病理特征的关系

*hOGG1*启动子区域-1096 bp T/C多态（rs159153），该位点杂合子在中国人群中的报道频率为22.2%（www.HapMap.org），与本研究所测得频率22.5%无差异，不能说明该位点与NSCLC的发生有关，但对临床资料用*Fisher*确切概率法统计结果显示该位点与TNM分期情况相关，32例Ⅰ期+Ⅱ期患者中有4例杂合子，8例Ⅲ期+Ⅳ期患者中有5例杂合子（*P*=0.008），显示该多态与肿瘤的进程正相关，而与性别、年龄、组织学类型、吸烟史、肿瘤大小、淋巴结转移及其它均无统计学差异（表 2）。SNP位点-23A/G（rs1801129）没有报道频率，本研究的频率为2.5%。SNP位点-18G/T（rs1801126）在中国人群中的报道频率为4.8%，与本研究所得频率5%没有差异，Ishida等^[8]^曾报道日本人的该位点与肺腺癌明显相关（OR=3.15, *P*=0.014），而与肺鳞癌无明显相关（OR=0.86, *P*>0.05），由于样本量较少，本研究未做此具体分析。

**2:** 40例NSCLC患者中rs159153多态基因型与临床病理特征的关系 Associations between SNP rs159153 and clinical characteristics in 40 NSCLCs

Characteristic	Genotype		P^*^
	rs159153（T/T）	rs159153（T/C）	
Gender			1.000
Male	22	6	
Female	9	3	
Age (year)			0.476
≤60	16	6	
>60	15	3	
Smoking history			0.399
Yes	10	1	
No	21	8	
Histology			0.898
Adenocarcinoma	12	4	
Squamous cell carcinoma	13	3	
Other types	6	2	
Primary tumor			0.097
T1+T2	24	4	
T3+T4	7	5	
Lymph node OMs			0.712
N0	17	4	
N1+N2+N3	14	5	
TNM Stage			0.008
Ⅰ+Ⅱ	28	4	
Ⅲ+Ⅳ	3	5	
Total	31	9	
^*^Fisher exact test.			

### 2.3 NSCLC患者中临床病理特征与吸烟史的关系

本研究对这40例NSCLC患者的吸烟史调查表明，吸烟与NSCLC病理分型有关，11例有吸烟史的患者中只有1例是腺癌（*P*=0.027），这与以往的报道结果一致^[9]^。此外，吸烟史与淋巴结的转移情况也有关，21例无淋巴结转移的患者中有9例吸烟，19例有淋巴结转移的患者中只有2例吸烟（*P*=0.034）(表 3）。

**3:** 40例NSCLC患者中临床病理特征与吸烟史的关系 Associations between clinical characteristics and smoking history in 40 NSCLCs

Characteristic	Smoking history		P^*^
	Yes	No	
Gender			0.124
Male	10	18	
Female	1	11	
Age (year)			0.173
≤60	4	18	
>60	7	11	
Histology			0.027
Adenocarcinoma	1	15	
Other types	10	14	
Genotype			0.399
rs159153（T/T）	10	21	
rs159153（T/C）	1	8	
Primary tumor			0.254
T1+T2	6	22	
T3+T4	5	7	
Lymph node OMs			0.03.4
N0	9	12	
N1+N2+N3	2	17	
TNM Stage			0.080
Ⅰ+Ⅱ	11	21	
Ⅲ+Ⅳ	0	8	
Total	31	9	
^*^Fisher exact test.			

## 3 讨论

肺癌发病率、死亡率近年来呈上升趋势，80%的临床肺癌都是NSCLC。其病理分型分期直接与其诊治和预后相关，争取早期诊断在目前是治疗肺癌的关键，利用分子生物学手段对癌前病变或组织形态学仍处于正常时期的病例进行早期检测已成为一大热点。作为DNA切除修复酶基因，*hOGG1*在肿瘤发生中的作用已经有许多报道^[10-12]^。该基因与肺癌的关系已引起密切关注^[13-15]^。

本实验首次研究了该基因启动子区域的突变与NSCLC的关系，结果发现SNP位点rs159153与NSCLC的TNM分期相关，该位点杂合的患者肿瘤进展较快，预示着不良的预后。这一位点与肿瘤等疾病的相关性在国内外均未曾报道。由于该位点位于*hOGG1*基因的启动子区域，我们猜测其可能对该基因的表达效率有所影响。已有研究^[12]^发现*hOGG1*基因表达量的下降会影响DNA的修复，可能与部分肺癌的发生密切相关。*hOGG1*基因启动子区域CpG岛的甲基化、基因位点的杂合性缺失、基因突变及染色体的畸变等都可能是其表达量下降的原因。除了表达水平上的调节，其外显子的多态和选择性拼接可以形成多种类型的hOGG1蛋白，不同类型的蛋白对DNA修复能力也有所不同，其中第326位密码子C/G多态产生hOGG1-Ser326和hOGG1-Cys326两种蛋白，前者对DNA的修复能力比后者强，目前对该基因多态的研究多集中于此位点^[1, 7, 10, 14, 15]^。此外，hOGG1蛋白对氧化产物的识别能力可能也影响其在细胞中的定位，从而影响对DNA损伤的修复。最近Campalans等^[16]^研究发现，人细胞中的hOGG1蛋白原本定位于可溶性核质中，UVA照射后开始重新分布到核基质。可以观察到hOGG1蛋白集中定位在核骨架和细胞器官上，散布在与转录和RNA剪接相关的染色质区域。突变hOGG1蛋白不能与底物结合，显示hOGG1蛋白的重新定位不依赖于酶对DNA损伤的识别。研究同时发现，UVA照射后hOGG1蛋白到核骨架的补充可以被抗氧化剂阻断，提示反应性氧化产物是hOGG1蛋白重新分布的信号。

在本研究中发现吸烟者所致的NSCLC患者淋巴结转移进程明显较慢，而SNP位点rs159153杂合的患者却明显进程较快，显示遗传因素在NSCLC的发生发展过程中起着不可忽视的作用。可能其基因本身的缺陷使得DNA氧化损伤难以修复，而导致的突变如果发生在呼吸链相关基因，则可能会加剧DNA氧化损伤的发生。也可能烟草所含的致癌物主要是通过其它途径影响肿瘤的发生，我们的结果也显示吸烟与NSCLC的病理分型明显相关，而该位点多态与NSCLC的病理分型无关。

总之，肿瘤的发生和转移是一个多阶段、多基因参与的过程，单个基因改变不足以解释肿瘤发生的机制，许多肿瘤抑制基因都与肺癌的发生发展有关^[17]^。目前我们只是初步研究了该基因启动子区域的SNP位点rs159153与NSCLC的关系，该位点如何参与*hOGG1*基因表达的调控，与DNA上其它功能性位点之间的相互作用机制及其对hOGG1蛋白活性的影响还有待我们进一步深入研究。
